# Fluidic Patterning of Transparent Polymer Heaters

**DOI:** 10.1038/s41598-018-34538-w

**Published:** 2018-11-01

**Authors:** Laura J. Romasanta, Philip Schäfer, Jacques Leng

**Affiliations:** 1 University of Bordeaux, LOF, Solvay, CNRS, UMR 5258, Pessac, 33600 France; 2 University of Bordeaux, ISM, CNRS, UMR 5255, Talence, 33405 France

## Abstract

Semi-conducting polymers are promising materials for current and next generations of electronic devices, sensors and actuators, especially regarding their ability to conform to flexibles architectures. In particular, aqueous-based dispersions of semi-conducting complexes such as PEDOT:PSS can be printed using a variety of coating techniques and the conductivity of the final deposit may reach high values upon a proper treatment. The micro-structuration of these polymeric deposits remains challenging and of prime importance for further integration. We show here that a microfluidic post-treatment of PEDOT:PSS films of permits us to boost *locally only* their conductivity by several orders of magnitude, with a micron scale resolution. This is a fast process (~second), straightforward to upscale, that yields conductive patterns within the pristine film. Taking advantage of the localized Joule’s effect, we evidence using quantitative thermography a very efficient heating behaviour of the conductive tracks, which makes these polymeric structures promising candidates for low cost, clean-room free electrodes for lab-on-chip applications.

## Introduction

The actual quest for new materials with outstanding features in terms of electrical properties, transparency, stretchability, ability to generate or store energy, etc., is currently booming in perspective of portable, wearable, flexible, low energy consumption, low cost, and connected devices in a wealth of very different domains: healthcare^1–3^, energy^4–6^, communication^7,8^, to cite only a few. As key constructs in the fabrication of these devices, conductive structures—e.g., layers, lanes, interconnects—are always required and must sometimes be transparent^9–11^. Intrinsically conducting polymers comply with most of these requirements, and among them aqueous dispersions of PEDOT:PSS [poly (3,4-ethylenedioxythiophene), PEDOT, complexed with polystyrene sulphonic acid, PSS] are easily processed into thin films; the latter exhibit high ductility with however moderate conductivity, which can nevertheless be enhanced with secondary doping^12,13^. Extensive research has elucidated the role secondary dopants onto the molecular and mesoscopic conformation of PEDOT^14,15^, which may indeed result in organic, highly conducting coatings. Here, we present a simple yet powerful and versatile method that permits us to alter *locally only* PEDOT:PSS thin films in order to design conductive lanes on an initially barely conductive (pristine) PEDOT:PSS film deposited onto any type of substrate. More specifically, we contact a film with a liquid (secondary) dopant via a microfluidic masking tool. Where the film has been in contact with the dopant, its conductivity is largely enhanced while remaining barely conductive everywhere else. The method is fairly precise and simple, as opposed to sophisticated and ultra-precise lithographic and lift-off techniques^16^. Also, it is scalable using a variety of (possibly continuous) printing or masking tools but we limit our attention here to the basics of the local fluidic modification of a thin film.

## Results

**Figure 1:**
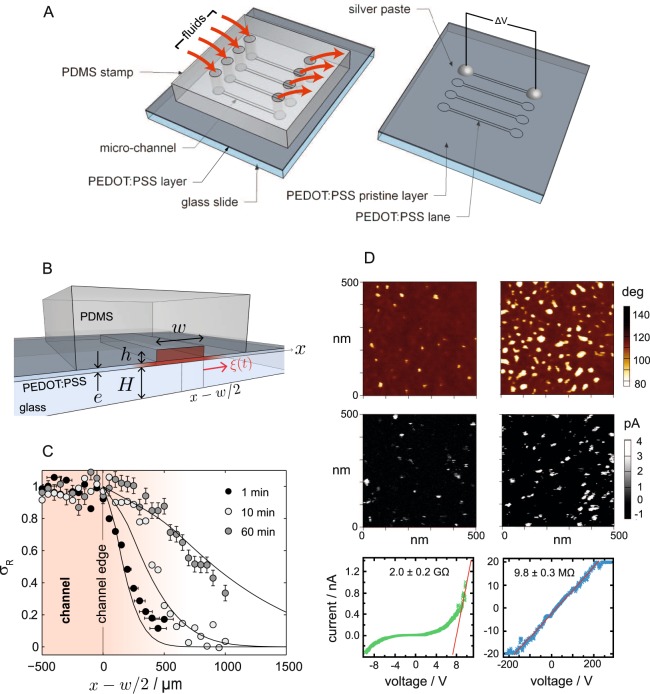
(**A**) Sketch of the microfluidic patterning technique. (**B**) Cross-section of the geometry with focus on one channel: typical dimensions are *w* = 1.5 mm, *h* ≈ 50 *μ*m, *e* ≈ 100–300 nm, *H* = 1 mm. The length of the channels is *L* = 1 cm. (**C**) Relative Raman red shift for thin films treated with ethylene glycol (EG) for different incubation times. The solid lines represent a diffusion-based model of EG in the thin film. (**D**) AFM characterization of the PEDOT:PSS film underneath (right column) and far (left column) from the microfluidic channel. From top to bottom: phase image, current map, and (exemplary) electrical behaviour of a single conductive spot.

The present process makes use of microfluidics in order to precisely control the contact in time and space of a so-called secondary dopant^17^ with the thin film: as sketched in Fig. 1A, this contact is achieved using a polydimethylsiloxane (PDMS) stamp at the bottom of which has been designed a set of arbitrarily shaped channels into which we can flow different fluids, e.g., secondary dopants (routinely called here the dopant). Upon contact with the film, the dopant diffuses into the thin film, in some cases plasticises it, and induces the conformational rearrangement of thiophene rings of the PEDOT chains, ultimately and locally boosting the film’s conductivity.

**Figure 2:**
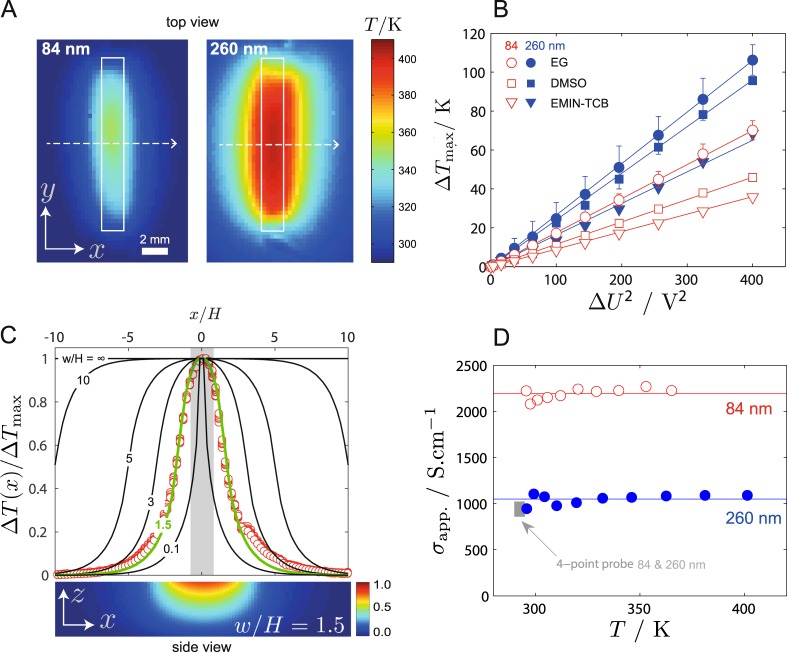
(**A**) QIRT images of virtual lanes undergoing Joule’s heating (EG patterning). The dimensions of the microchannel are shown in white and the dashed arrows indicate the temperature profiles shown in green on (**C**). (**B**) Temperature increase above room temperature against applied voltage, for lanes patterned with three different secondary dopants (EG: ethylene glycol^26,27^, DMSO: dimethyl sulfoxide^37–40^, EMIN-TCB: 1-ethyl-3-methylimidazolium tetracyanoborate^41,42^). (**C**) Top: surface temperature profiles in reduced units measured using QIRT (open red symbols, 84 nm-EG treated) and calculated for several aspect ratios *w*/*H* quoted on the figure; the grey region shows the spatial extent of the post treatment. Bottom: numerical model giving the temperature field in depth within the substrate below the heater. (**D**) Apparent conductivity of the lanes extracted from the thermal model (see SI).

Having several channels on the same stamp (Fig. 1A) allows us to quickly screen the efficiency of several secondary dopants. In best cases, patterning of virtual and tailored lanes leads to an increase of 2-to-3 orders of magnitude in conductivity as compared to the pristine layer. This can indeed be fully demonstrated by using quantitative infrared thermography (QIRT) which images the localized temperature increase (Fig. 2) due to the Joule’s effect when a voltage is applied along the virtual lane (Fig. 1A). Therefore, we not only show that this local patterning technique is fast and precise but we also quantify the ability of the conductive lane to locally generate heat. Thereby, QIRT turns out to be a very advantageous contactless method that yields a good estimate of the resistivity of the lanes, in fair agreement with the four-point probe measurements which are very difficult to perform on micron-sized transparent features. Eventually, we apply the patterning technique to design extremely efficient and cost-effective, solution-based micro-heaters for microfluidic applications^18^.

The cross-sectional view of the microfluidic geometry on Fig. 1B sketches the diffusion-based imbibition process of dopants from the microfluidic channel into the thin film, which will in turn alter its conductivity. The diffusion process involves two regimes: first, dopants diffuse vertically into the film, only where the latter is exposed to the liquid, right under the microfluidic channel; second, molecules diffuse in-plane under the PDMS stamp. For thin films (*e* < 300 nm), the first regime is fast and is followed by the second one, unsteady, whereby the dopant spreads within the film on a spatial extent $\xi (t) \sim \sqrt{Dt}$ outside the patterning zone (Fig. 1B,C). The cross-over between the two regimes occurs at $\tau  \sim {e}^{2}/D < {10}^{-2}$ s with *D* ≈ 10^−11^ m^2^s^−1^, a typical value for the diffusion coefficient of small molecules in a PEDOT:PSS matrix^19,20^.

Upon diffusion of these secondary dopants into the film, conformational changes of the PEDOT chains take place^21,22^, which are observable by use of Raman microspectroscopy. We incubate the thin films through the microfluidic geometry for a precise amount of time—from 1 minute to 1 hour—and rinse and anneal the films in order to arrest any further conformational reconfiguration of PEDOT chains. A pointwise mapping of Raman spectra perpendicular to the main channel shows that the vibrational band of PEDOT’s thiophene rings redshifts from *σ*_0_ ≈ 1438 cm^−1^ in the pristine film (far from the microfluidic channel) to *σ*_*T*_ ≈ 1434 cm^−1^ in the treated region (underneath the channel), indicating the dopant-induced transition from the benzoid to the quinoid form related to the coil to the expanded-coil conformation. The observed relative Raman redshift ${\sigma }_{R}(x)=[\sigma (x)-{\sigma }_{T}]/[{\sigma }_{0}-{\sigma }_{T}]$ is a function of the coordinate *x* perpendicular to the edge of the patterning channel (Fig. 1C), and varies from 1 all over the treated zone to 0 far away from it. The typical diffusion length $\xi (t)$ strongly depends on the incubation time and we found we could map the Raman redshift with the concentration field of a dopant which would diffuse in plane (solid line on Fig. 1C). This one-to-one mapping strongly suggests that the PEDOT conformational change is correlated to the presence of a dopant; additionally, the diffusion kinetics is best described with a concentration-dependent diffusion coefficient, consistent with a plasticizing effect of PSS matrix that is believed to enhance these conformational changes^23^. Eventually, the diffusion length $\xi$ permits us to define the spatial resolution of the current patterning technique: the shorter the treatment, the smaller $\xi$ and thus a superior definition. Flash post-treatments (≈sec) appear appealing in terms of up-scaling.

To show the fundamental reason for the conductivity increase, we correlate atomic force microscopy (AFM) maps of phase (intermittent contact mode) and current (contact mode) at two different yet registered positions (Fig. 1D), underneath and far from the microfluidic channels. It shows that the treatment has two main consequences: first, it opens up many low-phase regions which turn out to be PEDOT-rich grains, second, these grains show dissimilar I(V)-curves (see in Supplementary Information, SI, the full AFM characterization). In the pristine layer, PEDOT-rich grains display a diode-like behaviour while the post-treated region shows spots with an Ohmic response. It sustains a thorough reconfiguration of the film properties upon exposure to the secondary dopant, from PSS-insulated and disordered PEDOT grains toward a percolating network of conductive, PEDOT-rich and -reoriented grains^21,24,25^.

## Application

We now focus on the resulting conductive lanes working as heaters via Joule effect when a current is supplied to the lanes (Fig. 2B). Quantitative infrared thermography (QIRT) permits us to evidence a local release of heat at the level of the patterned lanes (Fig. 2A) with a temperature increase above room temperature which follows ${\rm{\Delta }}{T}_{{\rm{\max }}}=T-{T}_{0}\propto {\rm{\Delta }}{U}^{2}$ (Fig. 2B, with *T* measured at steady state in the middle of each lane). Not only does it fully corroborate the local boost of the thin film’s conductivity upon microfluidic patterning with a secondary dopant, but it also opens an interesting screening strategy for the dopants. Indeed, it is clear from Fig. 2B that not all dopants work the same, and among the limited set of secondary dopants we tested here, ethylene glycol is definitely the most efficient conductivity booster^26,27^, ultimately leading to a temperature increase up to 100 K.

The ability of a lane to develop a specific surface temperature relies on an interplay between three main factors: its heating power, the thermal properties of the environment, primarily the nature of the substrate it is laid on (thermally conducting or insulating), and its geometry especially for narrow lanes ($w/H\lesssim 1$) for which heat flow diverges significantly. For instance, a fairly conductive substrate such as glass acts as a heat sink whereas air is a good insulator; as a consequence, heat flows within the glass, and if the lane is not very wide, 3D diffusion into the thick substrate matters. It explains for instance why the temperature footprint seen from above the substrate with QIRT appears significantly larger than the actual patterned conducting lane (Fig. 2A,C). Conversely, in order to boost the heating efficiency of the lanes, the best would be to use an insulating substrate such as a polymer. We illustrate such an interplay in a generic and exact analytical model (see SI), which in turn permits us to perfectly fit the actual experimental surface temperature profile of the lane (Fig. 2C) *with a single free parameter*: the heating power density of the film (*q* in Wm^−3^) which is unknown and actually depends on the post-treatment. We extract *q* and translate it into an electrical conductivity *σ*_app_ (Fig. 2D) in excellent agreement with the 4-point probe measurements, at least for the thickest film. Surprisingly, the apparent conductivity also varies with film thickness *e*, a result which might be related with the dimensionality of the film whose structure evolves from a 2D to a 3D percolating network of PEDOT-rich grains as the thickness increases from ≈80 to 260 nm.

**Figure 3:**
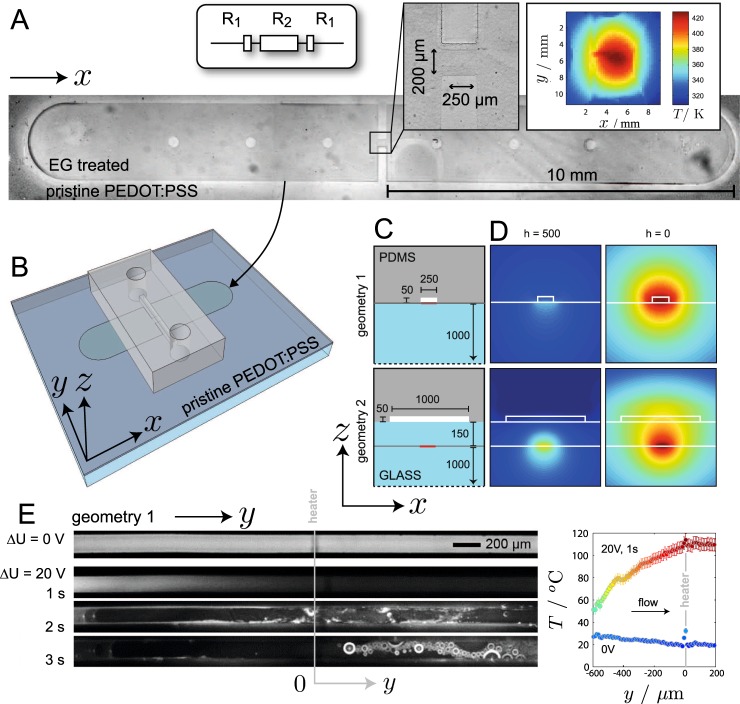
(**A**) Phase-contrast optical micrograph of *μ*-heater and design of the constriction following an electrical analogy. (**B**) 3D sketch of the microfluidic device including flow cell and *μ*-heater. (**C**) Two geometries with the fluidic channel either in direct contact (geometry 1) or with a spacer (geometry 2), dimensions in *μ*m. (**D**) Cross-sectional views of the temperature field calculated inside the microsystem, for the two geometries and for two different conditions: strong flow (*h* = 500 W.m^−2^.K^−1^, left) or stationary liquid (*h* = 0 W.m^−2^.K^−1^, right). Temperatures are scaled to the maximum that can be achieved in case of geometry 1 with no flow. (**E**) Left: fluorescence images of a rhodamine solution flowing at 100 *μ*L.h^−1^ without (upper one) and with heating power (Δ*U* = 20 V) supplied to the heater; Right: temperature profiles obtained from fluorescence imaging along the channel before and 1 second after switching on the power.

Our model yields robust engineering rules for optimizing the heating efficiency of any design of conducting lanes. Here, we take full benefit of these rules to integrate a low-cost and efficient micro-heater (*μ*-heater) within a microfluidic device. The *μ*-heater is designed by electrical analogy where resistors (the conductive lanes) are connected in series (Fig. 3A); assuming a constant sheet resistance *R*_*s*_ throughout any post-treated lane, the resistance of a local resistor reduces to its aspect ratio ${R}_{i}={R}_{s}({L}_{i}/{W}_{i})$ with *L*_*i*_ and *W*_*i*_ the length and width the i*th* resistor respectively, leading to ${\rm{\Delta }}T\propto {R}_{i}\propto {W}_{i}^{-1}$, at constant current. The design in Fig. 3A is patterned with a stamp which features a constriction that becomes the most resistive zone ${R}_{2}\ll {R}_{1}$ and acts as a localized *μ*-heater which can deliver of up to 420 K with a 84 nm-thin layer of PEDOT:PSS. Then, upon embedding the *μ*-heater into a mm-to-cm thick device yields a point source of heat (Fig. 3B,C).

Now, we stick right onto the *μ*-heater a PDMS channel (Fig. 3) in which we flow a rhodamine aqueous solution at a given rate. Fluorescence of rhodamine is known to be temperature-dependent and works here as a temperature probe^28^. We used two geometries (Fig. 3C): with the first one, a microfluidic PDMS channel is stuck directly onto the conductive film at the level of the *μ*-heater whereas in the second one, a thin glass layer is inserted in between the *μ*-heater and the fluidic channel in order to protect the polymeric *μ*-heater from direct contact with fluids. Figure 3D shows cross-sectional temperature fields calculated around the *μ*-heater in these two cases with realistic thermal properties and geometries for all constituents and for two very different conditions: the stationary liquid or a forced convection in the flow channel. These calculations show that heating can be quite strong even with a glass layer that spaces out the heat source from the liquid. Of course, the heating performance strongly depends on the flow rate which can, in extreme cases, lead to a complete convection of the heat by the liquid. From an experimental viewpoint, geometry 1 is the most efficient and quick-responsive one and we achieve very fast heating and subsequent boiling of water (Fig. 3E, water over-heated at ≈380 K in a few seconds while being injected at ≈298 K). However, geometry 1 is prone to degradation and we show in SI that passivation (geometry 2, Fig. 3C) is adequate for long term use, making the microfluidic chip re-usable and robust.

## Conclusion

We showed that it is possible to modify locally only the conductivity of a PEDOT:PSS thin film in order to print conductive patterns with a micron-scale resolution. Altogether, this patterning technique opens several perspectives: first, the local patterning of conductive zones in thin films of PEDOT:PSS can be widely tuned with, for instance, continuous printing or patterning techniques possibly on any type of substrate including flexible ones (also realized on PET, not shown here). Up-scaling is therefore possible in terms of substrate size and we forsee for instance the fabrication of transparent windows defoggers obtained by (dip?) coating followed with a localized post-treatment via screen, jet, off-set printing. On the small scale, the integration of conductive constructs—such as electrodes or heaters—into micro-devices seems straightforward and could be extended to 3D structures; as the method requires no clean room facility, it could lower significantly their costs of fabrication. Besides, the present method can be used as such to screen secondary dopants: instead of a few channels only (Fig. 1), it seems easy to implement tens to hundreds of flow channels in which many different dopants can be tested and, probably more interesting throughput-wise, mixtures and formulation of liquid dopants. Eventually, the method we developed for assessing the heating power of a thin film using QIRT can be used as such to quantify the performances of the many different formulations of conductive films that appear nowadays in literature^29–36^.

## Methods

PEDOT:PSS aqueous dispersion (PH1000) was purchased from Heraeus. Three different secondary dopants were tested and used as received: ethylene glycol (EG, anhydrous, 99.8%, Sigma Aldrich), dimethyl sulfoxide (DMSO, ACS reagent ≥ 99.9%, Sigma Aldrich), and 1-ethyl-3-methylimidazolium tetracyanoborate (EMIM TCB, ultrapure grade, EMD chemicals). For the fabrication of the elastomeric microstamp Sylgard 184 (Dow Corning) was used. Single-layer PEDOT:PSS thin films were obtained by spin coating 12.5 *μ*L.cm^−2^ of the aqueous dispersion at 1000 rpm for 60 s onto glass substrates previously cleaned by sonication in acetone, ethanol and water and pre-treated in air plasma for 10 min to enhance wettability. Thicker samples were made by repeating the spin coating process (with 25.0 *μ*L.cm^−2^) multiple times for the number of PEDOT:PSS layers needed (2 or 8 layers). All the thin films were dried in an oven at 120 °C for 15 min between each spin-coating deposition. PEDOT:PSS dispersion was sonicated for 1 h and filtered through a 0.45 *μ*m syringe filter prior to spin coating process.

PEDOT:PSS thin films were locally modified by using a PDMS-based stamp perfusion technique (see Fig. 1) where the stamps were fabricated using standard soft photolithography techniques. The stamp consists of a PDMS slab with several molded micro-channels (height = 50 *μ*m, width = 1500 *μ*m, length = 1 cm), each of them equipped with an inlet and an outlet (holes punched in the PDMS matrix) for introducing and recovering the fluids (secondary dopants), respectively. The PDMS stamp was carefully deposited onto the PEDOT:PSS film after treating it with a short exposure (1 minute) to air plasma in order to enhance adhesion with the thin film and thereby preventing fluid leakage. Each channel inlet was connected to a syringe needle via PFTE tubing. After filling the micro-channels, fluids were let to diffuse through the PEDOT:PSS thin film for a fixed amount of time (from ≈1 minute to 1 hour). Then, fluids were removed through the outlet by gently pushing air into the micro-channel, dried at 120 °C for 15 min, then rinsed several times with DI water to remove possible traces of secondary dopants on the film surface and again dried at 120 °C for 15 min. The elastomeric stamp was finally peeled off from the PEDOT:PSS thin film.

Thin films thickness measurements were carried out with a mechanical profilometer (AlphaStep D-500 Stylus Profiler). The sheet resistance of both PEDOT:PSS-based thin films was measured by using a four-point-probe consisting of four equally spaced test probes connected to a DC power supply (Keithley 2400). On each sample, at least three measurements were performed and averaged.

Raman spectroscopic measurements were recorded using a confocal Raman microscope (HR800, HORIBA Jobin Yvon) with a 532 nm excitation laser, a  × 20 objective lens (NA = 0.25) and a grating of 1800 lines per millimetre grating (spatial and spectral resolution were around 2.5 *μ*m and 0.2 cm^−1^ respectively).

AFM and conductive AFM (c-AFM) images were obtained using an Asylum Research MFP3D instrument. Topography mapping was performed in alternating contact mode with tips from BudgetSensors (type Multi75E-G, 75 kHz, 3 N/m, Cr/Pt-coating), ContGB-G (13 kHz, 0.2 N/m^−1^, Au-coating) or Asylum Research (type AC240TS, 70 kHz, 1.7 Nm^−1^). In contact-mode and c-AFM the same tips from BudgetSensor with conductive overall-coating were used. The samples investigated with c-AFM were prepared on the basis of 260 nm-thick PEDOT:PSS films ongold covered glass substrates.

PEDOT:PSS conductive lanes obtained were connected with silver paint to a DC power supply (Keithley 2400) and a voltage ramp was applied between 0 and 20 volts, each step for a period of 90 seconds. The 2D temperature distribution images were captured in real time by a FLIR7000 infrared thermocamera (equipped with a MWIR 50 mm lens). Temperature calibration was performed using a thin (80 *μ*m diameter) K-type thermocouple connected to a digital thermometer and to the post-treated PEDOT:PSS channels using a thermal paste. All temperature measurements were performed at room temperature under a fume hood with low air extraction.

## Electronic supplementary material

Supplementary Information
