# Supplementary material for: Fluidic Patterning of Transparent Polymer Heaters
Source: Sci Rep. 2018 Nov 1;8:16227. doi: 10.1038/s41598-018-34538-w (PMC6212434; doi:10.1038/s41598-018-34538-w)
Supplement: Supplementary file 1 — Supplementary Information [file 41598_2018_34538_MOESM1_ESM.docx]

Supporting Information

**Fluidic Patterning of Transparent Polymer Heaters**

Laura J. ROMASANTA, Philip SCHÄFER, Jacques LENG*

**Direct correlation of the nanoscale mechanical properties and the nanoscale electric conductivity on the PEDOT:PSS surface**

Apart from the characterisation of the application presented in the main article, a few questions have raised concerning the fundamental understanding of the conductivity of PEDOT:PSS and its boost upon solvent post-treatment. These questions have been tackled in literature, but thorough conclusions have not been fully achieved yet. Therefore, we investigated the correlation of the PEDOT:PSS structure on the surface regarding mechanical properties by alternating contact AFM and the electrical conductivity by c-AFM to reach more clarity. The mechanical phase shift of the AFM-cantilever oscillation in intermittent contact mode has often been interpreted as softness of the underlying material, but in general, there are a number of possible influences on the phase shift, that includes mechanical properties but also electronic properties, like local charge density or charge mobility. Reproducible results and agreement with phase images from literature suggest an agglomeration of PEDOT in certain grains or areas, resulting in PEDOT-rich and PEDOT-poor grains. This separation can lead to different mechanical and electrostatic properties of the grains. Although different research groups have presented current maps of PEDOT:PSS-surfaces [1] and others have imaged its mechanical properties in non-contact or in intermittent contact-mode [2] demonstrating the chemical composition differences by AFM phase contrast [3], this is the first direct correlation of phase images and conductivity maps of the same area of a solvent post-treated PEDODT:PSS thin film. As already shown in **Figure 1D** the number of conducting spots is increased relative to untreated PEDOT:PSS and the conductive spots show linear Ohmic behaviour. The salient conducting spots identified in the current map of EG-treated PEDOT:PSS in Figure S1A were marked, grouped and then superposed to the mechanical phase image in Figure S1B to analyse the correlations. Most conducting spots coincide with low phase shift spots, although a slight drift is observed and the convolution of the tip geometry at the tip apex also influences the outcome of fine-structures in the images. The red circular markers reflect the size of the used tip apex approximately. The trend is anyhow remarkable. The low phase shift regions correlate in a convincing manner with the conductive regions and therefore have to be interpreted as the PEDOT-rich grains connected with a penetrating conductive route through the thin film. Surely, not all low-phase shift spots show high conductivity (Figure S1). This is obvious since not all grains of high PEDOT content at the surface are well connected to a complete network that connects the respective surface grain with the gold-substrate. The areas of high density of conducting spots (Figure S1A, marked in blue ellipses) match well with the regions of high density of low-phase shift spots in the phase image (Figure S1A), confirming that under those PEDOT-rich surface areas a well-connected conductive PEDOT:PSS-network is present.


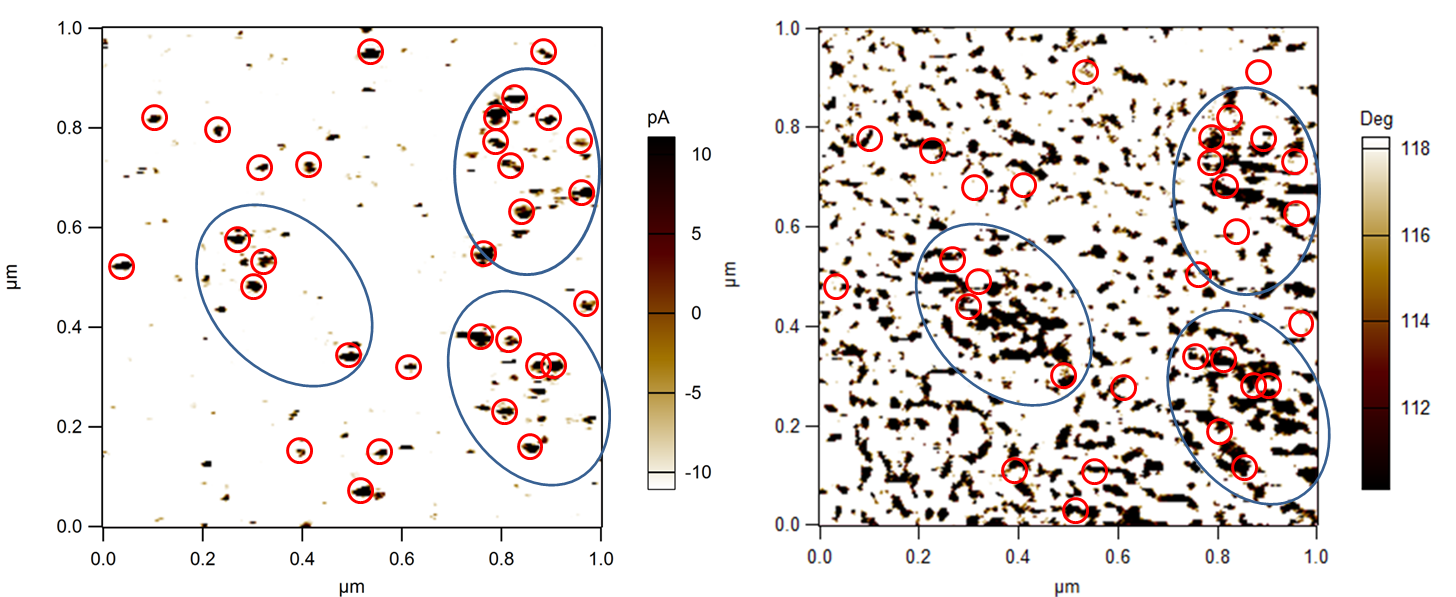


**Figure S1**. **A)** Current map of EG-treated PEDOT:PSS, conductive spots marked with a red circle correspond to **B)** marked spots of weak phase shift in a phase image of the exact same area, scanned in alternating-contact mode.

In addition, some conductive spots appear in areas that do not display a low phase shift. These conducting spots most likely appear because the high density of PEDOT-rich grains are covered by thin insulating layers of PSS in accordance with an established model depicted by J- P. Thomas *et al.* [4]. Only with increased electric fields, hole-tunneling [5] can occur through theses layers and strong currents appear beyond path-specific threshold voltages. This effect has been pushed to higher levels by L. S. C. Pingree *et al.* [6] by varying bias voltages.

Our experiment reveals a clear correlation of low phase shift spots and conductive pathways. It constitutes a key piece of the puzzle and connects several observations that explain the conductivity in these conductive polymer systems. We propose a distinct network of well-connected PEDOT-rich grains (**Figure S2**) induced by the treating solvent, in analogy to the description of H. Okuzaki *et al.* [7] and S. H. Chang *et al.* [8]. The connectivity is clearly enhanced by the treatment with solvents like EG, that causes phase separation between PEDOT and PSS resulting in PEDOT-rich grains, removal of PSS, and consequently good electrical connection points between PEDOT-rich grains, establishing a more distinct network of conductive paths through the film. This model is in agreement with conclusive investigations on this material system.


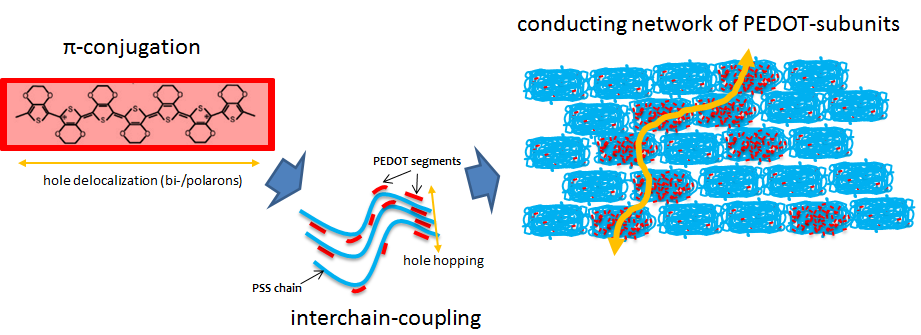


**Figure S2.** Left: molecular structure of PEDOT with delocalized holes, enabling polarons and bi-polarons. Middle: attachment of short PEDOT-chains and long PSS-chains with emphasis on good arrangement of PEDOT-segments for efficient hole-hopping. Right: assembly of PEDOT-rich PEDOT:PSS grains, eventually constituting a conductive path through the PEDOT:PSS thin film.

**Modelling the thermal behaviour of heaters**

We report on a simple model to account for the most crucial parameters involved in the thermal transfers of heaters. We focus first on the simple 1D geometry of a laminate (**Figure S3**): a homogeneous heating layer is in contact on one side with a fluid, and is laid on the other side onto a substrate. The thermal transfers in this geometry can be established analytically and give a simple overview of the most important parameters such as respective conductivities, thicknesses, etc.

We then limit ourselves to the thin film regime, where the heating layer is very thin as compared to its supporting substrate and we explore with numerical calculations the impact of the in-plane finite extent of a heating lane on its heating performances.


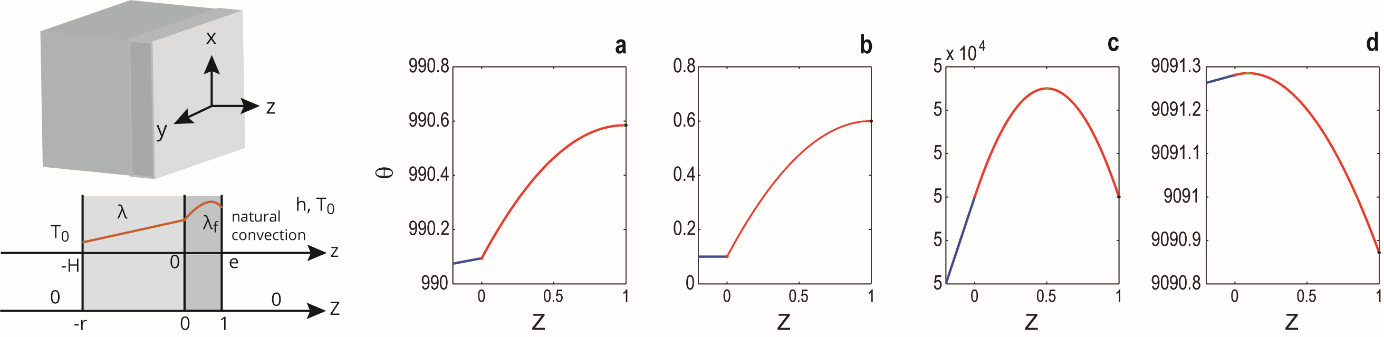


**Figure S3**. Left: 3D and projected view of the laminate geometry, infinite in the *x* and *y* directions and for which the thermal transfers reduce to the ones along the *z* direction. Right: reduced temperature profiles inside the film (red) and inside (part of) the substrate (blue) for several cases: a) Bi = 10^-5^; *η* = 10^-1^; *r* = 10^4^; b) Bi = 10^-5^; *η* = 10^-4^; *r* = 10^3^; c) Bi = 10^-5^; *η* = 1; *r* = 10^5^; d) Bi = 10^-4^; *η* = 1; *r* = 10^5^.

*Steady State for a Laminate.* We start with the laminate geometry given in Figure S3: the heating film (thickness *e,* thermal conductivity $\lambda_{f}$[W.m^-1^.K^-1^]) is deposited on a substrate (thickness *H,* thermal conductivity *λ*) with infinite extension perpendicular to the figure. Heat is generated into the film with a power density *q* [W.m^-3^] (generated for instance by Joule’s effect, *q* constant for $0<z<e$ and zero everywhere else). Heat is dissipated on the right side of the laminate (*z = e*) by thermal transfer due to natural conducto-convection in air (transfer coefficient *h* [W.m^-2^.K^-1^]) and on the left side of the film (*z = 0*) by conduction into the substrate. The latter is in contact with a thermal mass at *z = −H*, i.e., a large metallic body at room temperature *T_0_* [K]., *T(z = −H) = T_0_*.

Solving heat conservation permits us to find the temperature everywhere. In the substrate, $\lambda{\partial^{2}T}/{\partial z^{2}}=0$ with a temperature boundary condition *T(z=-H) = T_0_*. Heat flux at the substrate/film interface is conserved and gives another boundary condition: $\lambda{\partial T}/{\partial z|_{z=0^{-}}}=\lambda_{f}{\partial T}/{\partial z|_{z=0^{+}}}.$ In the film, $\lambda_{f}{\partial^{2}T}/{\partial z^{2}}+q=0$ with a flux boundary condition: $-\lambda_{f}{\partial T}/{\partial z|_{z=e}=h[T\left( e \right)-T_{0}]}$.

We introduce the following reduced variables: dimensions are given in units of film thickness, *Z = z/e*, and temperatures in units of *T_R_ = qe^2^/λ_f_*; the reduced temperature reads: *θ = (T − T_0_)/T_R_*. The ratios of thermal conductivities and thicknesses are defined as *η = λ_f_/λ* and *r = H/e* respectively. Eventually, we also introduce Biot number Bi*= he/λ_f_* which quantifies the efficiency of external to internal transfer mechanisms respective to the film. We note that the latter is always very small $\text{Bi}\approx{10}^{-5}$, meaning that temperature is nearly homogeneous within the film, and obtain an exact solution which can also be simplified:

$$\theta\left( Z \right)= -\frac{1}{2}Z^{2}+(Z+\eta r)\frac{2+\text{Bi}}{2[1+\text{Bi(1+}\eta r)]}\approx-\frac{1}{2}Z^{2}+\frac{Z+\eta r}{1+\text{Bi}\eta r}$$

in the film (*0 < Z < 1*) and

$$\theta\left( Z \right)=\eta(Z+r)\frac{2+\text{Bi}}{2[1+\text{Bi(1+}\eta r)]}\approx\frac{\eta(Z+r)}{1+\text{Bi}\eta r}$$

in the substrate (*-r < Z < 0*).

We explore some typical solutions for thin or thick, insulating or conducting substrates. In any case, we keep *e =* 100 nm as a reference thickness for the heating film, deposited on a thin (*H =* 100 µm) or ‘very’ thick (*H =*1 cm) substrate. It translates into an aspect ratio in the range *r*= 10^3^ − 10^5^. The transfer coefficient is due to natural convection and is also kept typical *h ≈* 10 W.m^-2^.K^-1^; the thermal conductivity of the film (typically a polymer) is also kept around *λ_f_  ≈*0.1 W.m^-1^.K^-1^ yet the thermal conductivity of the substrate may vary from 0.1 (polymer), to 1 (glass) to 10^2^ W.m^-1^.K^-1^ (metal). Therefore, *η* ranges in 10^-4^ − 1.

The ‘real’ case of 1 mm-thick glass substrate with natural convection (Bi = 10^-5^; *η* = 0.1; *r* = 10^4^) is shown in Figure S3a. *θ* reaches 10^3^ with no gradient on the right side because natural convection is not able to extract heat as compared to the heat sink role of the substrate. Maximum temperature is reached at the surface of the film, *Z* = 1 with *θ*(1) ≈ *ηr*(1 − Bi*ηr*) ≈ 0.99*ηr* = 990 (indeed very close to the exact value given in Figure S3a). This temperature does not depend on the film properties (except for its thickness) and temperature gradients across the film are very small. This is our most important result in terms of properties of thin polymeric heaters: heat flows in the substrate only, not in air, and the temperature increase is maximum at the film surface and directly proportional to the heat source: *T(e) – T_0_ = qeH/λ* in real units.

Other cases are instructive as well. Figure S3b shows the case of a thin and conducting substrate (Bi = 10^-5^; *η* = 10^-4^; *r* = 10^3^). The thin film delivers all the power to the substrate owing a very small thermal resistivity of the latter.

On the opposite, Figure S3c shows how a very thick insulating substrate (Bi = 10^-5^; *η* = 1; *r* = 10^5^) permits the film to reach its maximum temperature *θ*(1) ≈ *ηr*/2 = 5 10^4^.

Eventually, we show in Figure S3d the case of a thick and insulating substrate and forced convection (Bi = 10^-4^; *η* = 1; *r* = 10^5^). Here, heat is also dissipated at the upper surface due to strong convection and we find that θ(1) ≈ *ηr/*(1 + Bi*ηr*) ≈ 9091, in excellent agreement with exact calculations.

*Finite Size Effects.* The previous description holds for infinite films and gives an overview of the important parameters that control the local increase of temperature. In particular, the case of a thin film onto a 1 mm-thick glass substrate and in contact with air shows that the latter is acting as a perfect insulator and the temperature increase scales like *ΔT = qeH/λ*.

Now, we take into account the finite-extent of conductive tracks in the plane of the substrate (*x, y* Figure S3) by introducing a lateral and finite spatial width *w* of the lane in the *x* direction while keeping it infinite in the *y* direction. The heat transfers are solved in the (*x, z*) plane with a numerical solver (pdetool, Matlab) and with the following assumptions, also shown in **Figure S4**: the heating track is infinitely thin; we neglect transfers into air and set a no-flux boundary condition at *z* = 0; the bottom side of the substrate is kept at room temperature.


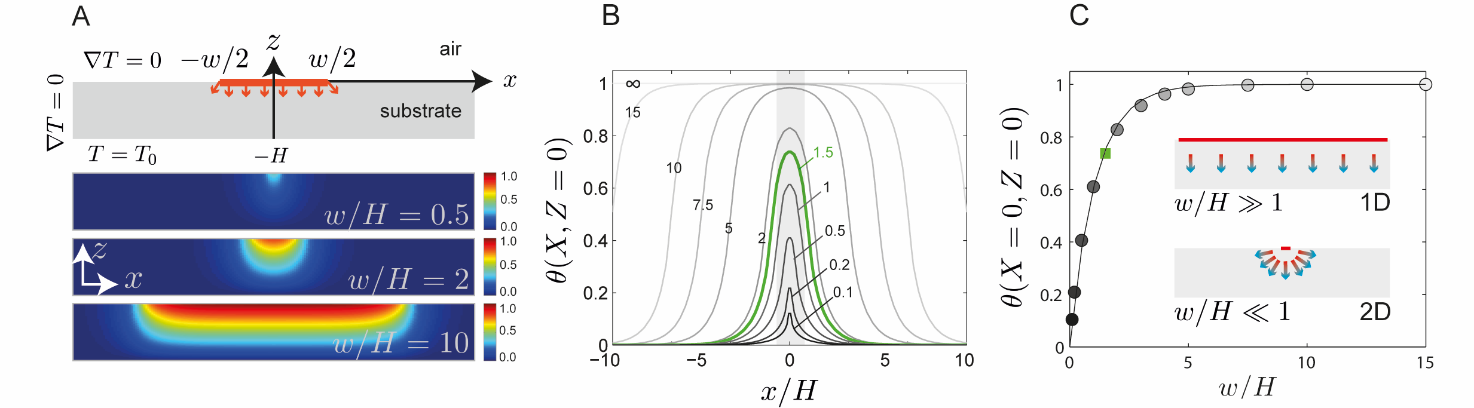


**Figure S4**. A) Scheme and boundary conditions for solving the 2D heat transfer problem, with 3 examples for different aspect ratio *w/H* of the heating track; B) Reduced temperature along the upper surface for several aspect ratios given in the figure; C) Reduced temperature in the middle of the track against aspect ratio (insert: transition from a 1D to a 2D geometry as a function of the aspect ratio of the track). In B and C the green line and symbol correspond to the experimental case.

We obtain the temperature everywhere in the substrate and plot in Figure S4b the surface temperature in reduced units for many aspect ratios *w/H* of the lane. We recover that for a near infinite lane ($w\gg H$) the maximum temperature reaches *θ* ≈ 1, and for narrower tracks, finite size effects tend to diminish the maximum temperature the heater can deliver; it is due to the diverging aspect of the temperature field (Figure S4a, bottom) and also explains the footprint of the surface temperature that can exceed by far the size of the lane. Indeed, when $w\ll H$up to $w\sim H$, the diffusion of heat is set by a 2D transport and limited by the size of the substrate, even if a sharp hot spot can develop (Figure S4b). Eventually, the maximum temperature that can be reached is well fitted by an empirical law $\theta_{\text{max}}=1-exp(-w/H/1.1)$ (Figure S4c).

*Link with Experiments*. In the experimental case, *w/H* = 1.5, and therefore $\theta_{\text{max}}\approx0.75$ which then relates the temperature increase to material properties:

${\Delta T}_{\text{max}}\approx0.75\left( T-T_{0} \right)= 0.75{\Delta U}^{2}\left( \frac{H}{\lambda} \right)\left( \frac{\sigma e}{L^{2}} \right)$,

where we assumed heating *via* Joule’s effect from a film with a uniform conductivity *σ*. Beside the absolute value of the temperature increase, the spatial profile is found to be perfectly described by the numerical model, see Figure 2C.

**
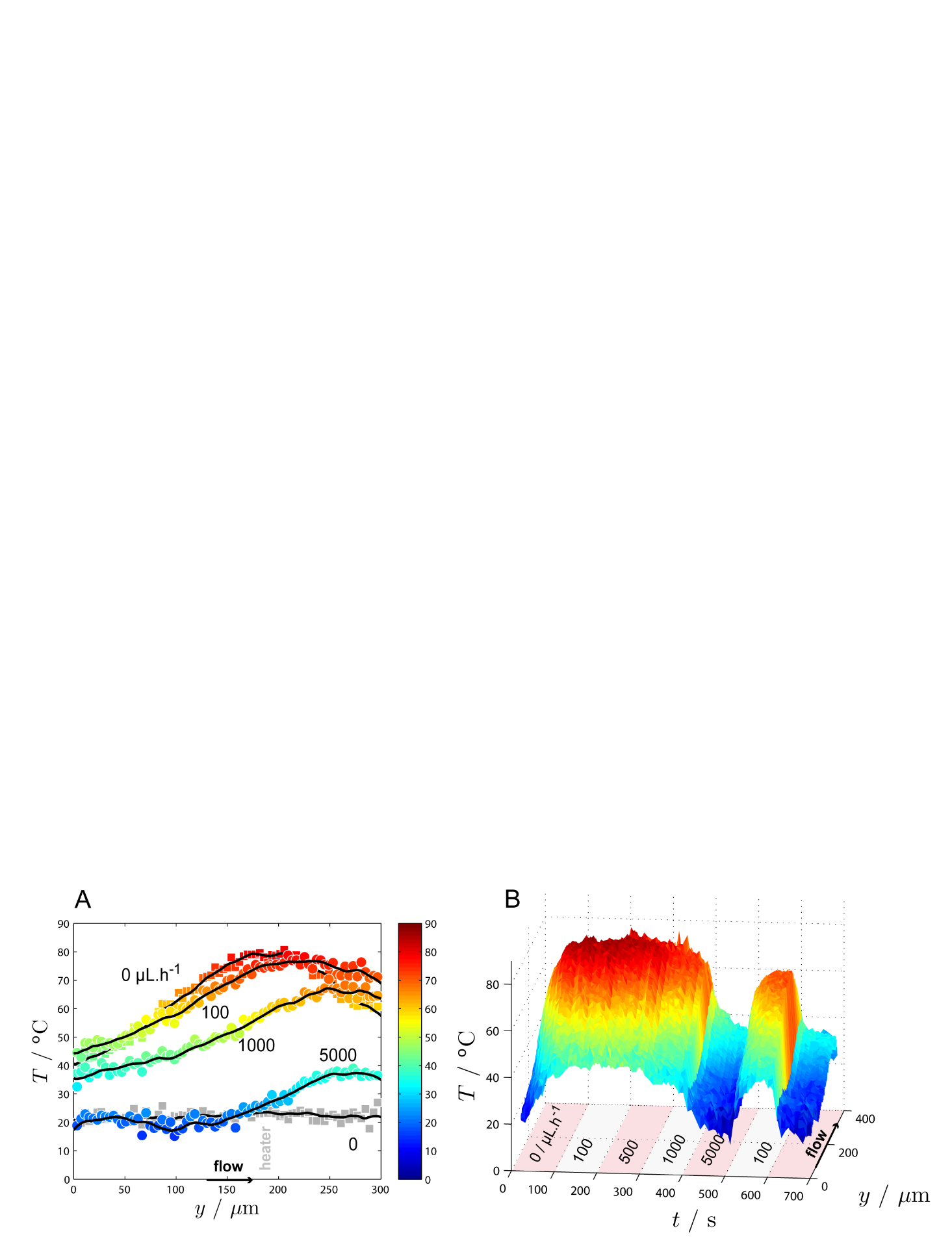
**

**Figure S5.** A) Temperature profiles along the channel for geometry 2 (see Figure 3C) where the flow microchannel is separated (and passivated) from the microfluidic heater by a 170-µm thick glass spacer, for several flow rates of water dyed with rhodamine (grey symbols show the temperature profile at rest). B) Sequence of several flow rates and corresponding temperature profiles showing a fairly quick response of the embedded temperature-sensitive probe (≈ 50 s).

**Microfluidic heaters**

We extend this model in order to design microfluidic heaters and to understand the role of flow. The sketch of Figure 3D shows the geometry used in the model, very close to the ones we used in experiments, and material properties such as thermal conductivities (glass and PDMS) are taken into account. An out-of-plane thermal transfer coefficient is introduced in order to mimic the effect of flow, even if we acknowledge that a better solution should involve the Poiseuille-Hagen type of flow. Yet, neglecting it, we can map the effect of flow onto *h* and find that at rest (*h* = 0), the planar micro-heater behaves roughly as a point source whereas at high flow rate (high *h*), heat is taken away with the flow. Also, using a glass spacer in order to passivate the polymeric heater remains efficient. With such geometry, it is possible to use for a long time the heater and we demonstrate in **Figure S5A** that the flow rate has indeed a major effect on the temperature profile. We can also extract the kinetics for reaching this steady state, typically less than 1 minute, in agreement with the thermal transient dictated by the geometry, say $\tau\sim h^{2}/D_{T}$ where *h* is a typical thickness and $D_{T}=\lambda/\rho C_{P}$the diffusion coefficient of heat in the material. For 1 mm-thin glass substrate or a 5 mm thick PDMS slab, $\tau\sim1-{10}^{2}$ s.

**Supporting references**

[1] L. S. C. Pingree, B. A. MacLeod, D. S. Ginger, *J. Phys. Chem. C* **2008**, *112*, 7922–7927; X.-D. Dang, M. Dante, T.-Q. Nguyen, *Appl. Phys. Lett.* **2008**, *93*, 241911.; H. Okuzaki, Y. Harashina, H. Yan, *Eur. Polym. J.* **2009**, *45*, 256–261.

[2] T.-R. Chou, S.-H. Chen, Y.-T. Chiang, Y.-T. Lin, C.-Y. Chao, *J. Mater. Chem. C* **2015**, *3*, 3760–3766.; J. P. Thomas, L. Zhao, D. McGillivray, K. T. Leung, *J. Mater. Chem. A* **2014**, *2*, 2383–2389., D. Alemu, H.-Y. Wei, K.-C. Ho, C.-W. Chu, *Energy Environ. Sci.* **2012**, *5*, 9662–9671.

[3] X. Crispin, F. L. E. Jakobsson, A. Crispin, P. C. M. Grim, P. Andersson, A. Volodin, C. van Haesendonck, M. Van der Auweraer, W. R. Salaneck, M. Berggren, *Chem. Mater.* **2006**, *18*, 4354–4360.

[4] J. P. Thomas, L. Zhao, D. McGillivray, K. T. Leung, *J. Mater. Chem. A* **2014**, *2*, 2383–2389.

[5] X. N. Xie, K. K. Lee, X. Chen, K. P. Loh, C. H. Sow, A. T. S. Wee, Org. Electron. 2010, 11, 1432–1438.

[6] L. S. C. Pingree, B. A. MacLeod, D. S. Ginger, *J. Phys. Chem. C* **2008**, *112*, 7922–7927

[7] H. Okuzaki, Y. Harashina, H. Yan, *Eur. Polym. J.* **2009**, *45*, 256–261.

[8] S. H. Chang, C. H. Chiang, F. S. Kao, C. L. Tien, C. G. Wu, *IEEE Photonics J.* **2014**, *6*, 1–7.
